# Increasing Antiradical Activity of Polyphenols from Lotus Seed Epicarp by Probiotic Bacteria Bioconversion

**DOI:** 10.3390/molecules23102667

**Published:** 2018-10-17

**Authors:** Ying Liu, Xuekuan Hui, Salam A. Ibrahim, Wen Huang

**Affiliations:** 1 College of Food Science and Technology, Huazhong Agricultural University, Wuhan 430070, Hubei, China; yingliu@mail.hzau.edu.cn; 2 College of Food Science and Engineering, Shandong Agriculture and Engineering University, Jinan 250100, Shandong, China; zhuzaitian8@163.com; 3 Department of Family and Consumer Sciences, North Carolina A&T State University, 171 Carver Hall, Greensboro, NC 27411, USA; ibrah001@ncat.edu

**Keywords:** polyphenols, lotus seed epicarp, probiotic bacteria, antioxidant activity, bioconversion

## Abstract

Probiotic bacteria is able to metabolize polyphenols and produce functional compounds. In this study, we investigated the ability of probiotic bacteria including *Lactobacillus*, bifidobacteria and *Enterococcus* strains to increase the antioxidant capacity of polyphenols from lotus seed epicarp (PLSE) at full ripening stage. The results showed that the six selected strains of probiotic bacteria grew well in De Man, Rogosa and Sharpe (MRS) broth with PLSE, and their resistant extent to PLSE varied from strain to strain. The metabolized PLSE was found to have good antioxidant properties on 3-ethylbenzothiazoline-6-sulfonic acid (ABTS^+^) and 1,1-diphenyl-2-picryl-hydrazyl (DPPH) radicals in vitro. Five polyphenol compounds—chlorogenic acid, caffeic acid, catechin, epicatechin and hyperoside—were suggested as the major bioactive metabolism for the antiradical activity of PLSE metabolized by *Lactobacillus reuteri* DSM20016, *Enterococcus faecalis* M74 and *Bifidobacterium breve* ATCC 15701. Moreover, *L. reuteri* DSM20016 and *E. faecalis* M74 were found to have a high PLSE bioconversion rate. Our results suggested that both *L. reuteri* DSM20016 and *E. faecalis* M74 might have excellent potential for the bioconversion of PLSE to increase its antiradical activity.

## 1. Introduction

*Nelumbo nucifera Gaertn*, also known as lotus, is an important aquatic economic crop to be cultivated in Asia, Oceania and America for more than 3000 years and used as a medicinal herb in eastern Asia [1,2]. Lotus seeds and leaves have multiple medicinal uses as antiemetic, anticancer, antiviral, inflammatory and antiobesity remedies [3,4,5]. It has been reported that lotus flowers had a long history in folk medicine and were used for the treatment of hematemesis, eczema and stomach ailments [6,7]. The pharmacological activities of lotus are closely related to the abundant metabolites found in almost all of its tissues. These metabolites include alkaloids, steroids, flavonoids, triterpenoids, glycosides and polyphenols [8,9,10,11].

Thus far, several kinds of bioactive compounds have been obtained from lotus seed epicarp. It was reported that some glycosylated flavonols and aglycone flavonols were isolated and identified from fresh lotus seed epicarp [12,13]. Previously, we identified epicatechin, hyperoside, catechin and isoquercitrin from polyphenols of lotus seed epicarp (PLSE). Polyphenols of lotus seed epicarp reportedly have good antioxidant properties including reducing power, 3-ethylbenzothiazoline-6-sulfonic acid (ABTS^+^) radical scavenging activity and 1,1-diphenyl-2-picryl-hydrazyl (DPPH) radical scavenging activity [14]. Polyphenols from medicinal plants have also attracted a great deal of attention because of their significant antioxidative activities. However, most polyphenols were found to be not absorbed in their native forms, the polyphenols must be modified by microbial conversion [15]. It has been reported that *Lactobacillus* metabolized polyphenols based on glycosidase [16]. Researchers have reported that grape seed polyphenols were converted to gallic acid, pyrogallol, and catechol by the glycosidase of *Lactobacillus plantarum* [17]. Therefore, probiotic bacteria bioconversion would play a major role in enhancement of the antioxidant activities of polyphenols [18,19].

There have been few studies related to the capacity of probiotic bacteria, such as *Lactobacillus*, bifidobacteria and *Enterococcus* strains, to metabolize polyphenol compounds. However, there is no study about the effects of probiotics on the antioxidant activities of PLSE. Thus, the aim of this study was to define the ability of selected bacterial strains for increasing the antiradical activities of PLSE. The individual effect of PLSE on the growth of six strains of probiotic bacteria was also investigated.

## 2. Results and Discussion

### 2.1. Effect of PLSE on the Growth of Probiotic Bacteria

**Figure 1:**
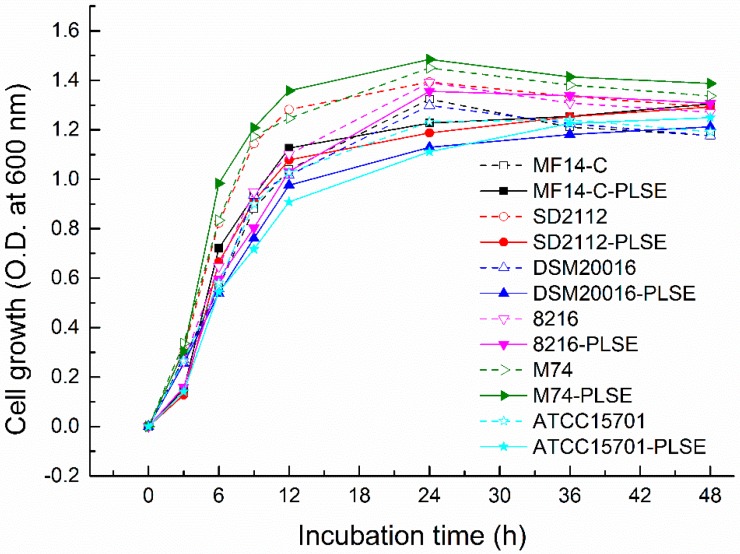
The cell growth of six strains of probiotic bacteria in De Man, Rogosa and Sharpe (MRS) broth (with or without polyphenols from lotus seed epicarp (PLSE)) for 48 h at 37 °C.

The bacterial cell growth of six selected bacterial strains in De Man, Rogosa and Sharpe (MRS) broth with or without PLSE for 48 h at 37 °C is shown in Figure 1. All of the six bacterial strains in the PLSE treated group showed a similar growth curve with those of control group without PLSE (Figure 1). Overall, *Enterococcus* exhibited a higher growth rate than those of *Lactobacillus* and bifidobacteria strains, and *Lactobacillus* had a higher growth rate than that of bifidobacterial. Similar results were also reported by other researchers, who observed that *Lactobacillus* had a higher population (1.41–1.65 OD_600 nm_) than those (1.18–1.32 OD_600 nm_) of bifidobacteria in MRS with *Pueraria flos* isoflavones [20]. Among the six selected strains, PLSE affected the growth rates of four strains including *L. reuteri* MF14-C, *L. reuteri* SD2112, *L. delbrueckii* 8216 and *B. breve* ATCC 15701 compared with the control group at the first 3 h of fermentation. The four strains in the control group showed a population of 0.264–0.307 OD_600 nm_ (Figure 1) while their population decreased to 0.128–0.16 OD_600 nm_ (Figure 1) in the PLSE treated group at the first 3 h of incubation. However, the growth rates of *L. reuteri* DSM20016 and *E. faecalis* M74 were not inhibited by the addition of PLSE. Generally, *E. faecalis* M74 showed the highest growth rate and *B. breve* ATCC15701 showed the lowest growth rate with or without PLSE. The *Lactobacillus* and *Enterococcus* strains exhibited a similar growth curve. The greatest exponential increase of the cell growth occurred over the first 12 h of incubation, and *E. faecalis* M74 showed the highest growth rate during the exponential period. Whereas the growth rate was reduced between 12 and 24 h of incubation. The results indicated that the cell growth of *Lactobacillus* and *Enterococcus* strains changed from the exponential to the stationary growth phase. The highest population of *Lactobacillus* and *Enterococcus* strains appeared at 24 h, and their bacterial growth cure showed the death phase appeared between 24 and 48 h in the control group. Similar results were also reported by other researchers in the study of polyphenols on lactic acid bacteria growth [17]. Compared to *Lactobacillus* and *Enterococcus* strains, *B. breve* ATCC15701 also showed a typical bacterial growth cure, while its highest population appeared at 36 h. However, highest population of *L. reuteri* MF14-C, *L. reuteri* SD2112, *L. reuteri* DSM20016 and *B. breve* ATCC15701 appeared at 48 h in the PLSE treated group, which indicated that PLSE prolonged the stationary growth phase of the four strains. Our results indicated that all strains tested grew well in MRS broth with PLSE, and their resistant extent to PLSE varied from strain to strain. It has also been reported that the growth of *Lactobacillus* was not inhibited by grape polyphenols, and grape polyphenols were effective as a growth-stimulatory factor for *Lactobacillus* [21].

### 2.2. ABTS^+^ Radical Scavenging Ability of Metabolized PLSE

**Figure 2:**
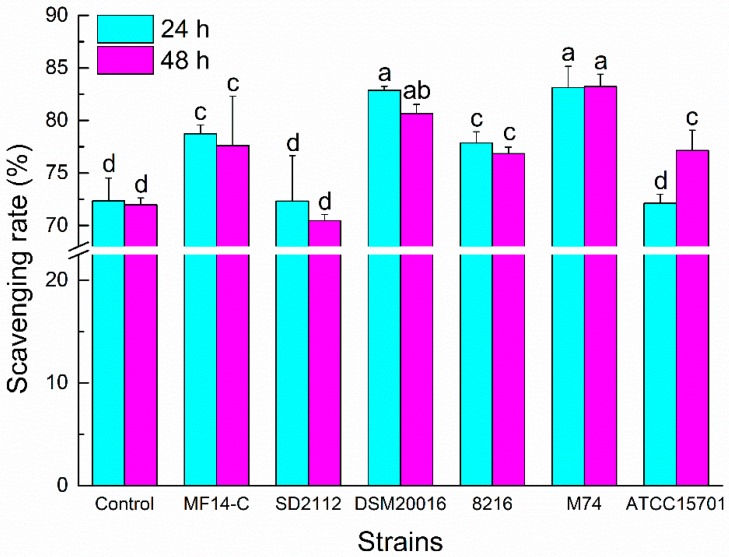
3-ethylbenzothiazoline-6-sulfonic acid (ABTS^+^) radical scavenging activity of PLSE fermented by six strains of probiotic bacteria at 37 °C for 24 h and 48 h. Different superscript small letters (a, b, c, and d) are significantly different (*P* < 0.05).

The ABTS^+^ radical is usually used to estimate the total antioxidant capacities of chemical compounds. As shown in Figure 2, the ABTS^+^ radical scavenging abilities of PLSE metabolized by *L. reuteri* MF14-C, *L. reuteri* DSM20016, *L. delbrueckii* 8216 and *E. faecalis* M74 increased compared with the control PLSE (*P* < 0.05) at 24 h of incubation. Whereas there was no increase for the scavenging abilities of PLSE fermented by *L. reuteri* SD2112 and *B. breve* ATCC 15701. Each strain except *L. reuteri* SD2112 had potential activity to increase the ABTS^+^ radical scavenging abilities of PLSE at 48 h of incubation. Generally, the ABTS^+^ radical scavenging abilities of metabolized PLSE varied depending on the type of probiotic bacteria. After 24 h of incubation, the ABTS^+^ radical scavenging abilities of PLSE metabolized by *Lactobacillus* (without *L. reuteri* SD2112) and *Enterococcus* were higher than that of bifidobacteria. The result suggested that *Lactobacillus* had a greater efficiency than bifidobacteria for the hydrolysis of PLSE. Liu et al. [20] also reported that *Lactobacillus* showed a greater efficiency than bifidobacteria for the hydrolysis of tectoridin. *Lactobacillus reuteri* SD2112 and *B. breve* ATCC15701 showed no effect on the antioxidant capacity of PLSE, the ABTS^+^ radical scavenging rate was not increased at 24 h of incubation. However, the ABTS^+^ radical scavenging ability of PLSE metabolized by *B. breve* ATCC15701 significantly increased (*P* < 0.05) at 48 h of incubation. This result might be caused by the release of some glycosidase from disrupted cells of *B. breve* ATCC15701 during 48 h of fermentation. It was notable that the ABTS^+^ radical scavenging rates of PLSE metabolized by *Lactobacillus* and *Enterococcus* strains no longer increased at 48 h of incubation. The results indicated that 24 h of fermentation was suitable for *Lactobacillus* and *Enterococcus* strains, while 48 h was good for bifidobacteria. A similar result was reported by Pyo et al. [22], who observed that *Bifidobacterium* spp. needed more time than that of *Lactobacillus* sp. for the bioconversion of the glucoside isoflavones. Our results suggested that *L. reuteri* DSM20016 and *E. faecalis* M74 would have the highest efficiency to increase the ABTS^+^ radical scavenging ability of PLSE. These different results might be attributed to the varied activities of glycosidase in the selected probiotic bacteria.

### 2.3. DPPH Radical Scavenging Ability of Metabolized PLSE

**Figure 3:**
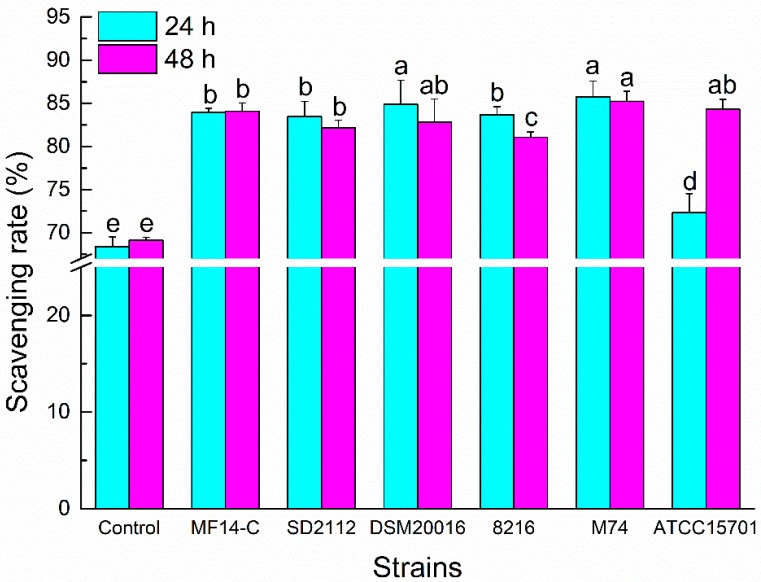
1,1-diphenyl-2-picryl-hydrazyl (DPPH) radical scavenging activity of PLSE fermented by six strains of probiotic bacteria at 37 °C for 24 h and 48 h. Different superscript small letters (a, b, c, d, and e) are significantly different (*P* < 0.05).

The DPPH radical is typically for the assay of free radical scavenging activities of varied antioxidants [14,23]. The DPPH radical scavenging rates of different PLSE samples are shown in Figure 3. Compared to the control sample, the DPPH radical scavenging rates of metabolized PLSE increased dramatically after the fermentation of the six selected bacterial strains (*P* < 0.05). After 24 h of incubation, the DPPH radical scavenging abilities of PLSE metabolized by *Lactobacillus* and *Enterococcus* were higher than that of bifidobacteria. *Bifidobacterium breve* ATCC15701 showed no effect on the DPPH radical scavenging rate of PLSE after fermentation for 24 h. The results showed that PLSE metabolized by *L. reuteri* DSM20016 and *E. faecalis* M74 had the highest scavenging activities among the selected six bacterial strains, and the DPPH radical scavenging rate of PLSE increased to 84.86% and 85.76%, respectively. After 48 h of incubation, there was no increase of the DPPH radical scavenging capacities metabolized by *Lactobacillus* and *Enterococcus* strains, possibly because the probiotic bacteria cells entered the stationary phase and the glucosidase activity began to decrease. However, the DPPH radical scavenging rate fermented by *B. breve* ATCC15701 significantly increased from 72.34% to 84.32% (*P* < 0.05). Our results indicated that the greatest increase of DPPH radical scavenging rate of PLSE occurred in the first 24 h of fermentation for *Lactobacillus* and *Enterococcus* strains, whereas 48 h for *B. breve* ATCC15701.

Due to the limitation of a single antioxidant property test to reflect the antioxidant capacity of samples, both ABTS^+^ and DPPH radical scavenging capacities were used to investigate the antiradical activities of different PLSE samples. In this study, a significant increase in the antioxidant activity of PLSE fermented by different bacterial strains was observed. It has previously been reported that the antioxidant capacity of phenolic compounds metabolized by various probiotics would increase [24,25]. The PLSE metabolized by *L. reuteri* DSM20016 and *E. faecalis* M74 showed the strongest potential antioxidant activities among the selected probiotic bacteria. Our results suggested that the bioconversion of PLSE fermented by *Lactobacillus* and *Enterococcus* strains occurred during the first 24 h. The bioconversion of PLSE corresponded to the exponential growth phase of *Lactobacillus* and *Enterococcus*. Similar to what was reported in previous studies [19], the metabolism of procyanidins from the pericarp of *Litchi chinensis* mainly occurred in the exponential growth phase of probiotic bacteria. Thus, it is recommended that 24 h of fermentation is an adequate time frame for the optimum increase in the antioxidant activities of PLSE metabolized by *Lactobacillus* and *Enterococcus*, and 48 h is needed for bifidobacteria. In addition, our results support the idea that lotus consumption may yield functional products due to the increased antioxidant capacity of metabolized polyphenol compounds by probiotic bacteria.

### 2.4. HPLC Analysis of Metabolized PLSE

**Figure 4:**
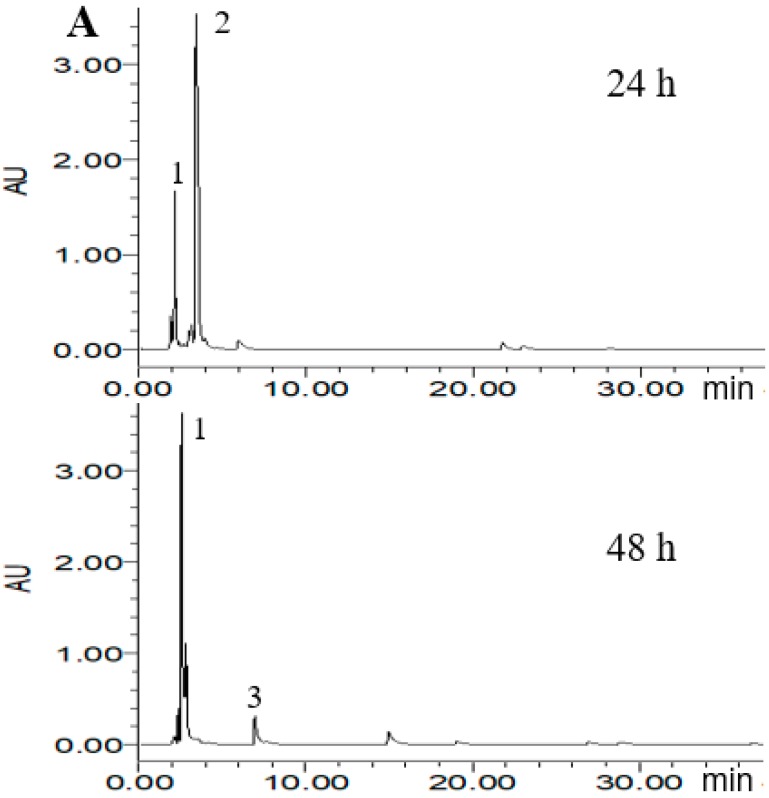

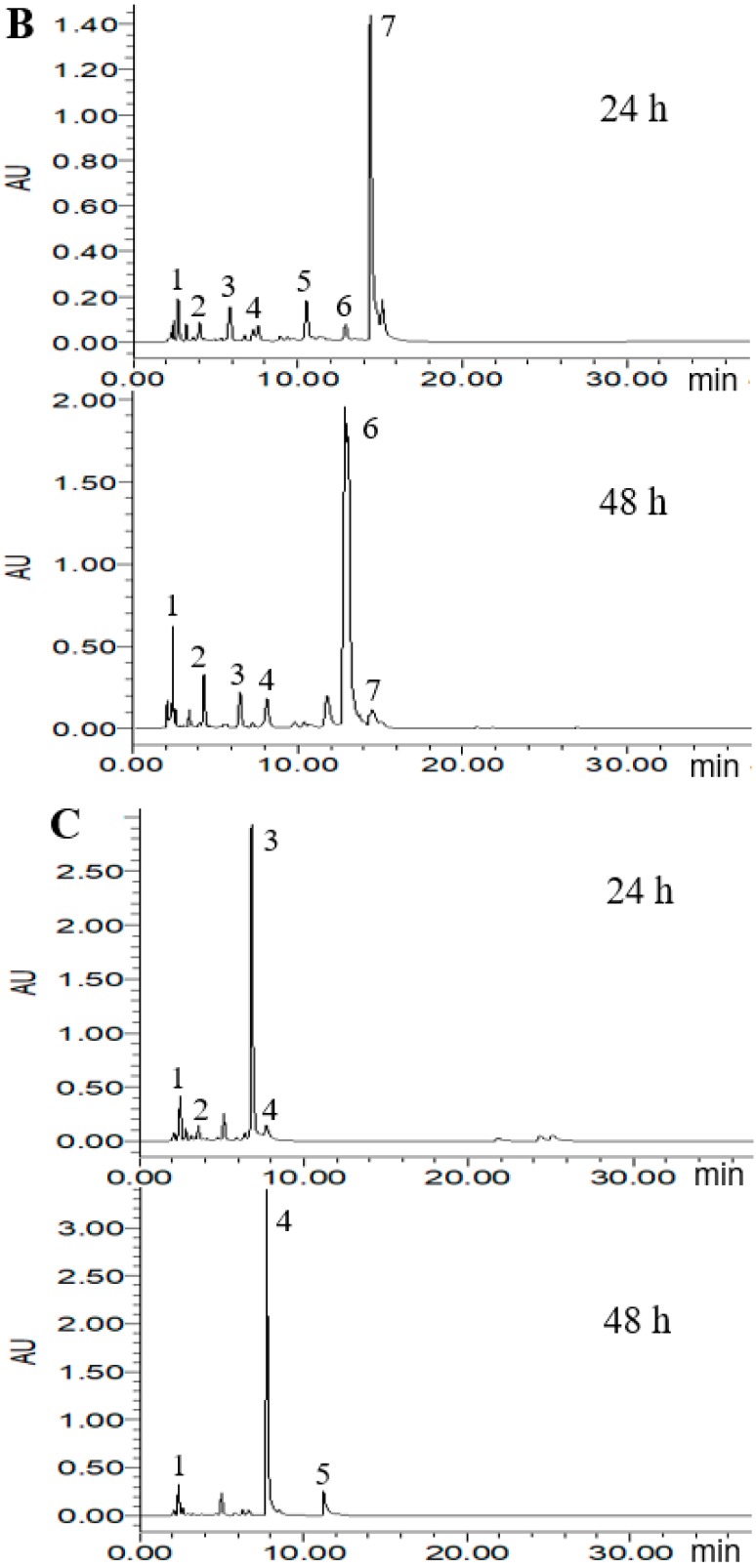

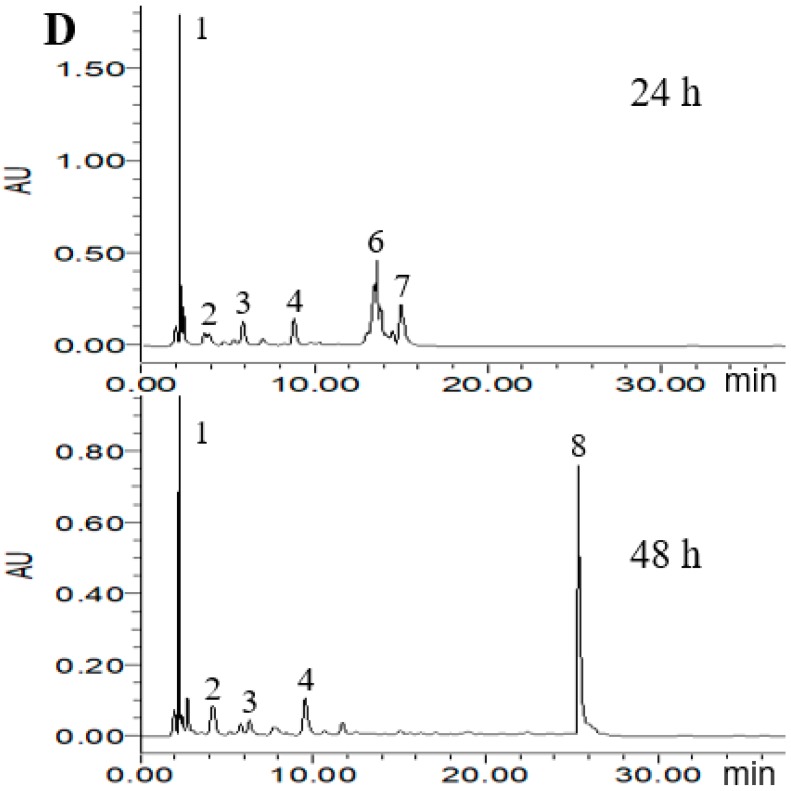
High Performance Liquid Chromatography (HPLC) chromatogram of PLSE fermented by probiotic bacteria at 37 °C for 24 h and 48 h. Control (**A**), *Lactobacillus reuteri* DSM20016 (**B**), *Enterococcus faecalis* M74 (**C**), *Bifidobacterium breve* ATCC 15701 (**D**). Peaks: 1, 2.2 min; 2, 3.8 min; 3, 6.3 min; 4, 8.2 min; 5, 10.9 min; 6, 13.3 min; 7, 14.7 min; 8, 25.7 min.

According to the ABTS^+^ and DPPH radical scavenging capacities experiment, three probiotic bacteria strains of *L. reuteri* DSM20016, *E. faecalis* M74 and *B. breve* ATCC15701 were considered to potentially increase the antioxidant activity of PLSE. Thus, High Performance Liquid Chromatography (HPLC) analysis was carried out to demonstrate that the three strains were able to metabolize PLSE. As shown in Figure 4, eight main polyphenol compounds were detected in the fermented samples by selected probiotic bacteria strains. In the chromatogram profiles obtained at 280 nm, the marked peaks 1–8 followed an elution order with the retention time of 2.2, 3.8, 6.3, 8.2, 10.9, 13.3, 14.7, and 25.7 min, respectively. As shown in Figure 4A, compounds 1 and 2 were found in the control group at 24 h of incubation and the concentration of compound 2 was higher than that of compound 1. After 48 h of incubation, compound 2 was not detected and compound 1 became the main polyphenol compound, another small peak (compound 3) was found in the control group. We have previously found that the control group at 24 h of incubation had the same antioxidant activity as that of 48 h (Figure 2 and Figure 3). It has been reported that the bioactivity was directly proportional to the concentration of the main metabolites [25,26]. Thus, our results suggested that compounds 1 and 2 were not the key compounds that affect the antiradical activity of PLSE. Compounds including 1, 2, 3, 4, 5, 6 and 7 were found in the sample fermented by *L. reuteri* DSM20016 at 24 h of incubation and compound 7 was the main polyphenol compound (Figure 4B). Whereas compound 6 became the main polyphenol compound after 48 h of incubation, and compound 5 was not found. *Lactobacillus reuteri* DSM20016 had the ability to increase the antioxidant activity of PLSE, which indicated that both compound 6 and compound 7 influenced the antiradical activity of PLSE. According to the reported HPLC results [19], compounds 6 and 7 were suggested to be catechin and epicatechin, respectively. In addition, *L. reuteri* DSM20016 at 24 h of incubation had similar antioxidant activity with that of 48 h (Figure 2 and Figure 3). Due to the similar antioxidant activity for catechin and epicatechin [27], our results suggested that catechin and epicatechin would play a comparable role for the antioxidant activity of metabolized PLSE. As shown in Figure 4C, compound 3 was the main polyphenol compound in the sample fermented by *E. faecalis* M74 at 24 h of incubation, and compounds 1, 2 and 4 were also detected. However, after 48 h of incubation, compound 4 changed to the main polyphenol compound, and compounds 1 and 5 were also found in this sample. According to the ability of *E. faecalis* M74 for increasing the antioxidant activity of PLSE, both compound 3 and compound 4 were suggested to affect the antioxidant activity of PLSE. As previously reported results by Sun et al. [28], compounds 3 and 4 might be chlorogenic acid and caffeic acid, respectively. Compounds including 1, 2, 3, 4, 6 and 7 were found in the sample fermented by *B. breve* ATCC15701 at 24 h of incubation and compound 1 was the main polyphenol compound (Figure 4D). This indicated that a small amount of PLSE was metabolized by *B. breve* ATCC15701 after 24 h of incubation. The results agreed that *B. breve* ATCC15701 was not able to increase the antiradical activity of PLSE compared with the control group at 24 h of incubation (Figure 2 and Figure 3). However, *B. breve* ATCC15701 had the ability to increase the antioxidant activity of PLSE after 48 h of incubation. A novel compound 8 was found to be the main polyphenol compound after 48 h of incubation. Compound 8 was suggested to be hyperoside, following the reported HPLC results [14,28]. Our results suggested that compound 8 influenced the antiradical activity of PLSE. In total, we found that five of the compounds, that is, chlorogenic acid, caffeic acid, catechin, epicatechin and hyperoside, would be the major metabolism of bioactive polyphenols for the antiradical activity of PLSE metabolized by selected probiotic bacteria.

## 3. Materials and Methods

### 3.1. Probiotic Bacteria and Growth Conditions

Three strains of *Lactobacillus reuteri* (MF14-C, DSM20016, and SD2112) and *Bifidobacterium breve* ATCC 15701 were obtained from North Carolina Agricultural & Technical State University (North Carolina, NC, USA). Strains of *Lactobacillus delbrueckii* 8216 and *Enterococcus faecalis* M74 were obtained from Huazhong Agricultural University (Wuhan, China). Before use, each individual bacterial colony strain was activated via separate inoculation into MRS broth in an anaerobic culture tube for 18 h at 37 °C.

### 3.2. Extraction of PLSE

Polyphenols from lotus seed epicarp at full ripening stage were extracted using our previously reported method [11]. The powder of lotus seed epicarp was homogenized in 60% of ethanol for 1 h at 50 °C. The extracted polyphenols were centrifuged at 10,000× *g* for 15 min by an Avanti^®^ J–E refrigerated centrifuge (Beckman Coulter, Brea, CA, USA). The resultant supernatant was evaporated by a rotary evaporator (RE-2000A, YaRong, Shanghai, China) and then lyophilized to obtain the PLSE.

### 3.3. Bacterial Activity on PLSE

*Lactobacillus reuteri* MF14-C, *L. reuteri* DSM20016, *L. reuteri* SD2112, *B. breve* ATCC 15701, *L. delbrueckii* 8216 and *E. faecalis* M74 were selected to study the effects of fermentation on the antioxidant activities of PLSE. Each sample of 200 mL of MRS broth containing 50 mg PLSE (30 mg polyphenols) was inoculated (1% *v*/*v*) with the six bacterial strains at 37 °C. The MRS broth containing 50 mg PLSE without bacteria incubated at 37 °C was the control group. The broth was then freeze-dried by a freeze drier (Betr 2–8 LD plus, Christ, Germany) after 24 h and 48 h of incubation. At last, each sample of 20 mL of 60% (*v*/*v*) aqueous ethanol was added to 1 g of lyophilized sample and shaken for 3 h to extract polyphenols from the fermented broth [19]. The separated supernatant was freeze-dried for further analysis.

An additional 200 mL of MRS broth containing 50 mg PLSE were inoculated (1% *v*/*v*) with the selected probiotic bacteria at 37 °C for 48 h to monitor cell growth by measuring the optical density (OD) at 600 nm wavelength during the incubation period. The MRS broth without PLSE inoculated (1% *v*/*v*) with the selected probiotic bacteria at 37 °C was the control group.

### 3.4. ABTS^+^ Radical Scavenging Activity

The ABTS^+^ radical scavenging activity of metabolized PLSE was measured by the reported method [29]. Briefly, the stock solution of ABTS^•+^ was taken and diluted to 50 mL before use. The absorbance of the diluent was measured at 734 nm to obtain the value of 0.7 ± 0.02. Next, the sample solutions of 2 mL were mixed with 4 mL of ABTS^•+^ diluent and shaken gently for 30 s. After that, the reaction was maintained at room temperature for 6 min. At last, the absorbance of the reaction was analyzed at 734 nm. The ABTS^•+^ scavenging activity was calculated as a scavenging rate (%) by the equation:

Scavenging rate (%) = [1 − (A_1_ − A_2_)/A_0_] × 100 (1)

Here A_0_ was the absorbance of the control (without sample), A_1_ was the absorbance of the mixture of sample and ABTS^•+^, and A_2_ was the absorbance of a sample blank (without ABTS^•+^).

### 3.5. DPPH Radical Scavenging Activity

The scavenging activity of metabolized PLSE on the DPPH was measured by the reported method with some modifications [30]. Each sample solution of 2 mL was first mixed with 2 mL of 0.2 mM DPPH• ethanol solution. Then the absorbance was measured against a blank at 517 nm after 30 min of reaction in darkness at room temperature. The DPPH radical scavenging activity was calculated as a scavenging rate (%) using the equation:

Scavenging rate (%) = [1 − (A_1_ − A_2_)/A_0_] × 100 (2)

Here A_0_ was the absorbance of the control (without sample), A_1_ was the absorbance of the mixture of sample and DPPH•, and A_2_ was the absorbance of the sample blank (without DPPH•).

### 3.6. HPLC analysis

The determination of PLSE was performed using a Waters e2695 HPLC system with a photodiode array detector and a C_18_ column (150 mm × 4.6 mm i.d., 5 µm, Shimadzu). The mobile phase was composed of 0.05% acetic acid in water (solvent A) and 0.05% acetic acid in acetonitrile (solvent B). The gradient elution procedure was as follows: 5% B to 35% B in 40 min, from 35 to 50% B in 10 min, from 50 to 80% B in 10 min and keep 10 min at 80% B. The flow rate was 1.0 mL/min and all the samples were detected at 280 nm.

### 3.7. Statistical Analysis

Results were expressed as mean ± standard deviation (SD). The analysis of variance was subjected to a one-way analysis of variance (ANOVA). The significant differences between the means of samples were determined by Duncan’s Multiple Range Test (*P* < 0.05).

## 4. Conclusions

In this study, we selected six strains of probiotic bacteria in order to metabolize PLSE to increase its antioxidant activity. Our results showed that the growth of the six strains of probiotics was not inhibited by PLSE during incubation of 48 h. Four of the strains—*L. reuteri* MF14-C, *L. reuteri* DSM20016, *L. delbrueckii* 8216 and *E. faecalis* M74—had the bioconversion capacity to increase the antioxidant activity of PLSE. We found that chlorogenic acid, caffeic acid, catechin, epicatechin and hyperoside would be the major bioactive metabolism that affected the antiradical activity of PLSE metabolized by *L. reuteri* DSM20016, *E. faecalis* M74 and *B. breve* ATCC 15701. Additionally, both *L. reuteri* DSM20016 and *E. faecalis* M74 had the highest bioconversion rate among the six bacterial strains. The bioconversion ability of probiotic bacteria for PLSE may have nutritional benefits due to the increase of antioxidant activity. Our results thus suggest that both *L. reuteri* DSM20016 and *E. faecalis* M74 could be applied to obtain bioactive polyphenols. It is notable that the bioactivity of polyphenols can be increased by the bioconversion of probiotics, and food polyphenols may induce a healthier intestinal flora profile. Thus, the intestinal health benefits of PLSE warrant additional research in this area.
